# Transcriptional Regulation of the Glutamate/GABA/Glutamine Cycle in Adult Glia Controls Motor Activity and Seizures in *Drosophila*

**DOI:** 10.1523/JNEUROSCI.1833-18.2019

**Published:** 2019-07-03

**Authors:** David Mazaud, Benjamin Kottler, Catarina Gonçalves-Pimentel, Sandra Proelss, Nadine Tüchler, Celine Deneubourg, Yoshihiro Yuasa, Celine Diebold, Heinz Jungbluth, Eric C. Lai, Frank Hirth, Angela Giangrande, Manolis Fanto

**Affiliations:** ^1^Department of Basic and Clinical Neuroscience, King's College London, SE5 9NU London, United Kingdom,; ^2^Institut de Génétique et de Biologie Moléculaire et Cellulaire, 67404 Illkirch, France,; ^3^Centre National de la Recherche Scientifique, UMR7104, Illkirch, France,; ^4^Institut National de la Santé et de la Recherche Médicale, U1258, Illkirch, France,; ^5^Université de Strasbourg, 67404 Illkirch, France, and; ^6^Department of Developmental Biology, Sloan-Kettering Institute, New York, New York 10065

**Keywords:** *Drosophila*, glia, glutamate/GABA/glutamine, repo

## Abstract

The fruitfly *Drosophila melanogaster* has been extensively used as a genetic model for the maintenance of nervous system's functions. Glial cells are of utmost importance in regulating the neuronal functions in the adult organism and in the progression of neurological pathologies. Through a microRNA-based screen in adult *Drosophila* glia, we uncovered the essential role of a major glia developmental determinant, *repo*, in the adult fly. Here, we report that Repo expression is continuously required in adult glia to transcriptionally regulate the highly conserved function of neurotransmitter recycling in both males and females. Transient loss of Repo dramatically shortens fly lifespan, triggers motor deficits, and increases the sensibility to seizures, partly due to the impairment of the glutamate/GABA/glutamine cycle. Our findings highlight the pivotal role of transcriptional regulation of genes involved in the glutamate/GABA/glutamine cycle in glia to control neurotransmitter levels in neurons and their behavioral output. The mechanism identified here in *Drosophila* exemplifies how adult functions can be modulated at the transcriptional level and suggest an active synchronized regulation of genes involved in the same pathway. The process of neurotransmitter recycling is of essential importance in human epileptic and psychiatric disorders and our findings may thus have important consequences for the understanding of the role that transcriptional regulation of neurotransmitter recycling in astrocytes has in human disease.

**SIGNIFICANCE STATEMENT** Glial cells are an essential support to neurons in adult life and have been involved in a number of neurological disorders. What controls the maintenance and modulation of glial functions in adult life is not fully characterized. Through a miR overexpression screen in adult glia in *Drosophila*, we identify an essential role in adult glia of *repo*, which directs glial differentiation during embryonic development. Repo levels modulate, via transcriptional regulation, the ability of glial cells to support neurons in the glutamate/GABA/glutamine cycle. This leads to significant abnormalities in motor behavior as assessed through a novel automated paradigm. Our work points to the importance of transcriptional regulation in adult glia for neurotransmitter recycling, a key process in several human neurological disorders.

## Introduction

Glial cells constitute a significant part of the nervous system and are devoted to a variety of functions of essential importance for the correct functioning of neurons, including a fundamental role in uptake and recycling of neurotransmitters (Rae et al., 2003). Therefore, glial cells remain of paramount importance throughout life to allow proper homeostasis of the nervous system, as evidenced by the association of glial functions' failures with neurological pathologies (Lynch et al., 2010; Furrer et al., 2011).

Glia in *Drosophila* provides many of the same functions as in mammals, including regulation of neurotransmitter uptake and recycling (Rival et al., 2004; Chaturvedi et al., 2014). We have performed a genetic screen to identify genetic elements required for glial function by specifically deregulating gene expression only in the adult *Drosophila* glia to avoid any developmental effect, and have used miRNAs to uncover key regulatory modules. The promiscuous nature of miRNAs allows to target and fine-tune the expression of several target genes (Lim et al., 2005; Bartel, 2009; Kozomara and Griffiths-Jones, 2011). In specific cell types and at specific time points, one or more of the miRNAs targets acquire special importance and can be singled out as the cause for the phenotype elicited by specific miRNAs (Silver et al., 2007; Bejarano et al., 2012).

Behavioral activity is the outcome of neural interactions mediated by different circuits. We hypothesized that glia manipulations will lead to a variety of behavioral consequences and structured our approach in three steps that allow detection of the progressive contribution of the genetic manipulations to different behavioral manifestations. First, we used fly lifespan as an unrefined readout. We then characterized endogenous and exogenous behavioral activity by simple motor readout (negative geotaxis) and, further, by adapting video-tracking methodology with control of mechanical stimulus. Using this novel approach allows detection of the progressive contribution of the genetic manipulations to different behavioral manifestations.

Here, we report that overexpression of miR-1 in adult glial cells shortens the fly lifespan, partially through the downregulation of Repo, a key transcription factor for the development of almost all *Drosophila* glia (Xiong et al., 1994; Halter et al., 1995). This led to the identification of a major regulatory module in *Drosophila* adult glial cell function maintenance, which points to the importance of transcriptional regulation of neurotransmitter recycling in glia with possible consequences for human neurological and psychiatric disorders.

## Materials and Methods

Drosophila *stocks and husbandry*. Flies were maintained at either 18°C or room temperature on standard cornmeal agar medium (0.8% w/v agar, 2% w/v cornmeal, 8% w/v glucose, 5% w/v Brewer's yeast, 1.5% v/v ethanol, 0.22% v/v methyl-4-hydroxybenzoate, 0.38% v/v propionic acid). All of the following lines were obtained from the Bloomington collection: *w*^1118^ (RRID:BDSC_3605), *repoGal4* (RRID:BDSC_7415), *dEAAT1Gal4* (RRID:BDSC_8849), *NP2222Gal4* (RRID:DGGR_112830), *MZ0709Gal4*, *moodyGal4*, *GMRGal4* (RRID:BDSC_9146), *Actin5cGal4* (RRID:BDSC_4414), *elavGal4* (RRID:BDSC_8765), *ubiGal80*^ts^, *tubGal80*^ts^ (RRID:BDSC_7019), *UAS-dEAAT1* (RRID:BDSC_8202), *UAS-iGluSnFRA184S* (RRID:BDSC_59610), and *UAS-repo*^IR^ *2* (TRiP.JF 02974 RRID:BDSC_28339). *alrmGal4* (RRID:BDSC_67031) and *UAS-Gat* were kindly provided by M. Freeman. *UAS-repo*^IR^ *1* (GD 10424, RRID:FlyBase_FBst0450092) was obtained from the VDRC collection, *UAS-repo-myc* was described previously (Matsuno et al., 2015), *UAS-repo* was also described previously (Yuasa et al., 2003), and *repo-nGFP* was generated by C. Diebold. *UAS-miR-1* was generated by E. Lai for the miRNA library (Bejarano et al., 2012). *UAS-EGFP-Gs2* was kindly provided by R.W. Ordway.

### 

#### 

##### Lifespan.

Lifespan analysis was performed as described previously (Nisoli et al., 2010). Briefly, all crosses were maintained at 18°C during the developmental stages of the progeny. Newly eclosed adult flies were collected within 5 d at 18°C. Females and males were pooled together and equally distributed within three vials. Sixty flies were assessed unless specified otherwise. All lifespan analysis was done in a controlled environment of 29°C and 60% humidity or 25°C when specifically stated. Using CO_2_ to anesthetize the live flies, those dead and alive were counted and live ones transferred into fresh vials three times per week. For the lifespans done with only 3 d at 29°C before being transferred to 18°C, flies were still counted 3 times per week but were transferred into a fresh vial only once a week.

##### Climbing assay.

Flies were collected from the same cross used for the lifespan experiment to assess their negative geotaxis reaction. Ten female flies from each genotype were transferred into 70 mm tubes. The tubes containing compared genotype were assessed at the same time using a custom-made array. Four sessions were recorded to evaluate the vertical position of each fly after 1 min. The scores from the 10 females were averaged for each repeat and the average of the four repeats calculated.

##### Automatic motor behavioral assay.

Single fly tracking was performed as described previously (Faville et al., 2015). In each experiment, ∼20 female flies per genotype were anesthetized on ice and individually placed into glass tubes. All of the genotypes were positioned on the same platform, with two shaftless motors placed under each subplatform containing each one genotype. The flies were allowed to recover for 30 min at 25°C before the start of the procedure. The protocol used for the stimuli response is presented with a schematic in Figure 7*A*. In summary, 6 stimuli events were equally split during a period of 2 h and 15 min, the first one starting after 30 min of recording and the last one 30 min before the end of the protocol. Each stimuli event was composed of five vibrations of 200 ms spaced by 500 ms. The *x*/*y* position of each single fly was tracked and analyzed using Drosophila ARousal Tracking (DART) software (Faville et al., 2015) to evaluate the relative speed and activity before, during and after the stimuli event. The speed analysis is used for the “stimuli response trace” and the general activity was used to deduce “active speed,” “mean bout length,” and “interbout interval” (for details, see Kottler et al., 2017) using a custom-made modification of the DART software (Faville et al., 2015). The DART-derived graphs were edited with Adobe Illustrator CC2017 (RRID:SCR_010279).

##### Heat-induced seizure assay.

The heat-induced seizure assay was adapted from one described previously (Sun et al., 2012). Ten flies per genotype were isolated into plastic vials with food 3 h before the assay. They were then allowed to accommodate into new plastic vials without food for 10–20 min before immersion in a 40°C water bath for 2 min. Each tube was video recorded during and after immersion and seizures were defined as a period of brief leg twitches and failure to maintain standing posture. The mean of the time to recover from seizure was calculated for each genotype. The experiment was repeated five times independently and averaged.

##### Adult brain staining.

Adult brain staining was performed as described previously (Baron et al., 2017). Briefly, flies were anesthetized on ice. Brains were dissected and put straight into 4% paraformaldehyde (PFA) for 45 min of fixation. They were then washed in phosphate buffer saline solution with 0.1% Tween (PBS-T) for 30 min before blocking in 5% bovine serum albumin (BSA) in PBS-T for 1 h at room temperature (RT) in a 96-well plate. Primary and secondary antibodies were diluted in blocking solution and incubated overnight at 4°C. After three washes of 15 min, brains were mounted in Vectashield on a slide surrounded by two coverslips on each side before being covered by another coverslip on top (to prevent the brains from being crushed). The antibodies were used as follows: anti-GFP (1/100, mouse, Roche, RRID:AB_390913), anti-GFP (1/500, chicken, kindly provided by M. Meyer), anti-Repo (1/100, mouse DSHB 8D12, RRID:AB_528448), anti-Elav (1/500, rat, DSHB 758A10, RRID:AB_528218 or 1/2000 mouse, DSHB, 9F8A9, RRID:AB_528217), and anti-GABA (1/1000, Sigma-Aldrich A2052, RRID:AB_477652 kindly provided by A. Delogu).

##### iGluSnFRA184S imaging.

Flies expressing the glutamate sensor iGluSnFRA184S were anesthetized on ice. The flies developed at 18°C and were transferred to 29°C for 7 d once they reached the adult stage. Brains were dissected into Schneider's medium and transferred directly into a dish filled with Schneider's medium to be imaged. Confocal pictures of each brain were taken within 20 min after dissection. *Z*-stacks of 1 μm sections were taken for each genotype. The microscope settings were established using control flies to have a GFP signal below saturation and kept unchanged throughout all acquisitions. These photographs were taken with a Nikon Spinning Disc confocal microscope and analyzed with ImageJ Fiji software (RRID:SCR_002285). A square of 166 × 166 pixels was drawn around the calyx region and the intensity was measured. The three highest values of each *z*-stack were averaged. At least three different calyx regions were measured per genotype.

##### TUNEL assay.

Fly brains were dissected in cold PBS and fixed for 45 min in 4% PFA at RT. After three washes of 10 min in PBS, the brains were incubated 10 min in 50 μl of proteinase K (20 μg/ml in PBS) and then washed twice for 10 min in PBS (to stop the permeabilization). For the positive control, a 10 min incubation in DNase I buffer was followed by a 10 min incubation in DNase I (7 U/ml in DNase I buffer) and then washed three times in double-distilled water (ddH_2_O). The brains for all the conditions were then immersed for 30 min in equilibration buffer and incubated 1 h at 37°C with a solution containing 44 μl of equilibration buffer, 5 μl of marked nucleotide mixture, and 1 μl of TdT enzyme (Promega). The reaction was stopped for 15 min in 2× SSC solution (diluted in ddH_2_O) and washed three times in PBS. The brains were then mounted in Vectashield (Vector Laboratories).

##### RNA extraction.

RNA was extracted as described previously (Napoletano et al., 2011). Briefly, 50 heads were cut and placed into a 1.5 ml microfuge tube followed by a snap freeze in liquid nitrogen. The heads were then homogenized in 100 μl of TriZOL (Invitrogen), after which an additional 200 μl of TriZOL was added. The mixture was incubated for 10 min at RT before adding 60 μl of chloroform. The tubes were vigorously mixed for 20 s and then incubated 2 min at RT before 15 min of centrifugation at 4°C and 12,000 rpm. Next, 200 μl of the aqueous phase (containing the RNA) was transferred into another 1.5 ml microfuge tube and 150 μl of isopropanol was added. A pellet of RNA appeared after 10 min of centrifugation at 4°C and 12,000 rpm. The pellet was washed three times with 75% EtOH and air-dried before the addition of 40 μl RNase-free ddH_2_O with 1 μl of Rnasin (Promega). The quality of the RNA extraction was assessed on a 1.5% agarose gel and the concentration and λ280/260 and λ260/230 ratios were measured with a Nanodrop.

##### qPCR.

A 1.5 μg sample of each RNA was sent to the King's College London Genomic Centre Facility for processing. qPCR was done using Universal Probe Library (UPL) probes and primers designed by the Universal Probe Library software (Roche) for quantification. Three independent biological replicates were measured for each genotype at each time point. *Gapdh* and *eIF4A* were used as controls. Details of oligos and UPL are given in Table 1.

**Table 1.** Oligos and UPLs used for qPCR

Gene	CG no.	UPL probe no.	Left oligo	Right oligo
*loco*	CG5248	116	CTGGTTTATCAACGCCTATGAA	GAGTGCGGAAGGAAGACTGT
*Gliotactin*	CG3903	69	CGAATCGTCCAATTACAGAGC	GAAAAATTCCAGGAGAAACTGG
*Gs2*	CG1743	127	GCACCCTCGACTTCATTCC	GCACGAGCTTCCATCGTAGT
*Pointed*	CG17077	63	CTTTCTGTCCAGCCTAGTTGAGT	TGCACAGATCCTTGCATCC
*dEAAT1*	CG3747	30	GAATAAATTTGCTTGACATCCTTTT	AAAGCACGATTGGCAGTCA
*wg*	CG4889	81	GGCAAAATCGTTGATCGAG	GCAGGACTCTATCGTTCCTTCA
*Gat*	CG1732	31	TTCTTTATGTCGATGAGAGCAGA	CCTTTCATATTGACTGACACAGTTG
*Gapdh*	CG12055	18	AAAAAGCTCCGGGAAAAGG	AATTCCGATCTTCGACATGG
*eIF4A*	CG9075	104	CGTGAAGCAGGAGAACTGG	CATCTCCTGGGTCAGTTGGT

Shown are sequences of oligos used for qPCR and probe numbers from the Universal Probe Library given for each gene tested.

##### Western blot.

An equal number of flies per genotype was decapitated with a scalpel, snap-frozen, and homogenized using a pestle in 1× sample buffer (0.05 mm Tris-HCl pH 6.8, 2.5% SDS, 10% glycerol, 0.0025% bromophenol blue) with 5% β-mercaptoethanol freshly added. The tube was centrifuged at 12,000 rpm at 4°C for 5 min. The supernatant was transferred into a new tube. The volume equivalent to five heads was loaded on 10% or 12% w/v polyacrylamide gel using a Bio-Rad gel electrophoresis apparatus. After separation in the gel, proteins were transferred onto a nitrocellulose blotting membrane (0.2 μm, Protran; GE Healthcare) for 1 h at 60 V and kept cold. The membrane was then blocked for 1 h in 5% BSA or 5% milk in TBS with Tween (TBS-T) at RT before incubation in primary antibody diluted in blocking buffer overnight at 4°C. The membrane was washed 3 times for 15 min in TBS-T at RT and then incubated 1 h at RT in secondary antibody. After three washes in TBS-T, the membrane was washed again in TBS. Enhanced chemiluminescence (ECL) reagent was mixed according to the manufacturer's instructions (SuperSignal WestPico or ECL Western blotting substrate; Pierce) and spread onto the membrane. After 2 min of incubation, the ECL liquid was removed and the membrane placed into a cassette with a film (Fujifilm) and developed. The film was then scanned and processed using Adobe Photoshop version 7.0.1 (RRID:SCR_014199) and the bands were quantified using Image Studio Lite version 4.0 software (RRID:SCR_013715). Antibodies were used as follows: anti-Repo (mouse, 1/200 in 5% BSA, DSHB 8D12, RRID:AB_528448), anti-Gat (rabbit, 1/10000 in 5% milk-TBS-T, RRID:AB_2569706, gift from Marc Freeman), anti-β-actin (rabbit, 1/3000 in 5% BSA, SAB 21338), anti-myc (mouse, 1/1000 in 5% BSA, Roche 9E10, RRID:AB_439694), and anti-GFP (mouse, 1/1000 in 5% BSA, Roche, RRID:AB_390913).

##### Statistical analysis.

All statistical analysis was performed with GraphPad Prism software (RRID:SCR_002798). For all lifespans, the statistical analysis was performed using the log–rank test of the Kaplan and Meier method. For the climbing assay in Figure 2*B* and behavioral experiments (DART), the statistical analysis was done by one-way ANOVA using Dunnett's multiple-comparisons *post hoc* test. For the analysis of experiments performed over time (climbing of Fig. 6*A*,*F*,*H*; seizure in Fig. 8*B*; and behavior in Fig. 8*A*) a two-way ANOVA was used with a Tukey's or Dunnett's multiple-comparisons test. All quantifications for Western blots and climbing assay with only two genotypes were analyzed at high stringency using a nonparametric Mann–Whitney test. The qPCR statistical analysis was performed using DataAssist software (RRID:SCR_014969) as a two-tailed *t* test. Significance is shown by asterisks in all figures as follows: *p < 0.05, **p < 0.01, ***p < 0.001, and ****p < 0.0001.

## Results

### miR overexpression screen in adult glial cells

**Figure 1.**
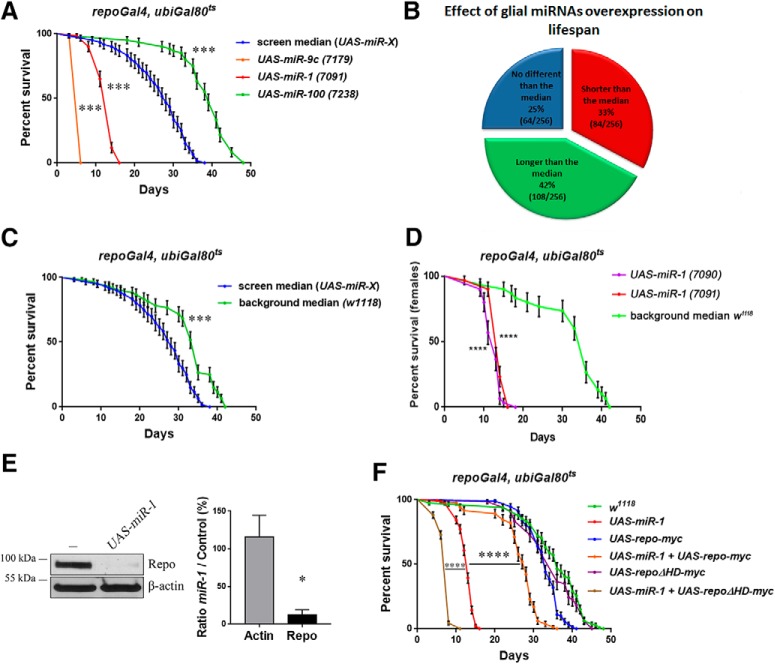
Effect of miRNA expression in adult *Drosophila* glia. All aging in this figure was performed at 29°C. ***A***, Examples of short-lived (*UAS-miR-9c* and *UAS-miR-1*) and long-lived (*UAS-miR-100*) miRNA lines overexpressed in adult glial cells compared with the screen median lifespan. Each lifespan was done with 60 flies (30 females and 30 males; see Materials and Methods). Log–rank (Mantel–Cox) test *p* < 0.000000000001 for all. For a full screen report, see Figure 1-1. ***B***, Summary of the miRNA screen. Each line is compared with the median lifespan of the library (256 lines from 133 miRNAs). ***C***, Comparison of the median from 6 *w*^1118^*; ubi-Gal80*^ts^/+*; repoGal4*/+ lifespan with the median lifespan of the screen *w*^1118^*; (ubi-Gal80*^ts^*; repoGal4)/ UAS-miR-X*. Log–rank (Mantel–Cox) test *p* < 0.0001 (***D***) Lifespan of female flies expressing *UAS-miR-1*, from an autosomal insertion (7091) and from an insertion on the X chromosome (7090) compared with female median *w*^1118^ controls. Both display a dramatic reduction in lifespan. Log–rank (Mantel–Cox) test *p* < 0.0001 for both. ***E***, Western blot analysis of Repo protein level upon miR-1 overexpression in adult glia. There is a strong reduction of Repo protein compared with control, quantified on the right panel (Mann–Whitney, *n* = 3, *p* = 0.049). The genotypes were *w*^1118^*; ubi-Gal80*^ts^/+*; repoGal4*/+ and *w*^1118^*; ubi-Gal80*^ts^/+*; repoGal4*/*UAS-miR-1* (7091). ***F***, Rescue of *UAS-miR-1* short lifespan by coexpression of *UAS-repo-myc* (log–rank Mantel–Cox test *p* < 0.000000000001), which by itself displays instead a mildly decreased lifespan compared with controls. Conversely, the coexpression of a mutated dominant-negative *repo* transgene, *UAS-repo*Δ*HD-myc*, enhanced the shortening of lifespan due to *miR-1* overexpression (log–rank Mantel–Cox test *p* < 0.0001), ruling out any weakening of the *UAS-miR-1* effect due to a second UAS-based transgene, whereas it had no effect of its own when expressed alone. The driver used was *repoGal4*, *ubi-Gal80*^ts^. Between 80 and 180 female flies were assessed. *p < 0.05, ***p < 0.001, ****p < 0.0001.

Using the *repoGal4*,*ubiGal80*^ts^ inducible system of expression, we have triggered expression in all adult fly glia of several *Drosophila* miRNAs from a library of >250 lines (Bejarano et al., 2012) and recovered a number of miRNAs that shorten or extend the fly lifespan compared with the screen median lifespan (Fig. 1*A*,*B* and Fig. 1-1).

10.1523/JNEUROSCI.1833-18.2019.f1-1 Figure 1-1 **miRNA-based screen in adult glia.** All lifespan analysis was performed at 29°C. 256 lines from 133 different miRNAs were overexpressed specifically in adult glia using *repoGal4* driver and the ubiquitous temperature sensitive *ubiGal80^ts^*. In green are the lines that significantly increase the lifespan from the library median lifespan and in red the ones that significantly decrease it. The miRNAs with all their assessed lines significantly different in the same direction are highlighted using the same colour code. Note that the *UAS-miR-1* line 7090 is inserted on the X chromosome, leading to expression only in females in our screen set up, explaining the low χ_i_² value in this Table. However, its effect is highly significant when considering only the female population, which expresses the transgene (Figure 1D). Download Figure 1-1, DOCX file

Although not isogenic, the library was generated in the *w*^1118^ background. We therefore ran a number of additional controls by crossing *repoGal4*,*ubiGal80*^ts^ to *w*^1118^ throughout the screen to compare their F1 with the median lifespan of our screen. Given the significant difference (Fig. 1*C*), we decided to focus on miRNAs that shorten lifespan because this class appears more robust compared with both controls.

### miR-1 expression in adult glia shortens the fly lifespan by repressing the expression of *repo*

Two independent *UAS-miR-1* insertions displayed a potent reduction in fly lifespan when expressed in the adult glia (Fig. 1*A*,*D* and Fig. 1-1). miR-1 is known to be expressed in the mesoderm during embryogenesis (Sokol and Ambros, 2005) and has been involved in setting up the repression of nonmuscle cell genes (Lim et al., 2005). In analyzing the predicted mRNA targets for miR-1 in the TargetScan and microRNA.org databases, we noticed that the pan-glial marker *repo* was predicted to be a target for miR-1. In agreement, specific adult expression of miR-1 in glia leads to a strong downregulation of the Repo protein expression in adult flies (Fig. 1*E*). We have reported previously that this effect is likely to be direct (Trébuchet et al., 2019). An exogenous *UAS-repo-myc* lacking the *repo* 3′-untranslated region and therefore resistant to repression by miR-1 (data not shown) significantly rescues the effect of miR-1 on lifespan (Fig. 1*F*). Conversely, a *UAS-repo*Δ*HD-myc* lacking the homeodomain failed to rescue the miR-1 lifespan phenotype (Fig. 1*F*), highlighting the requirement for a functional Repo protein and ruling out that the rescue by *UAS-repo* may be nonspecific due to the addition of a second UAS transgene.

### Repo is continuously required in adult glial cells for fly survival and motor behavior

**Figure 2.**
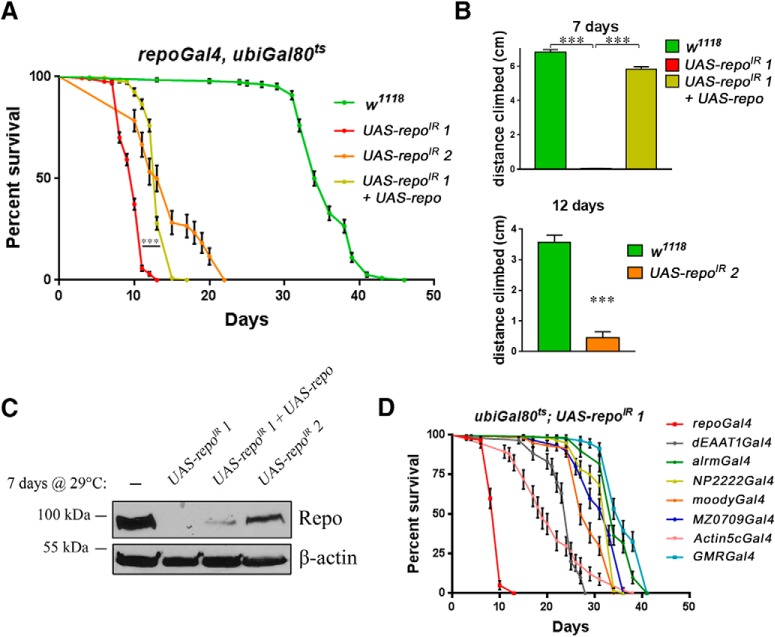
Repo protein levels influence *Drosophila* lifespan and behavior. All aging in this figure was performed at 29°C. ***A***, Lifespan of two different RNAi lines against *repo* and the rescue of the RNAi 1 by coexpression of a *UAS-repo* transgene (log–rank Mantel–Cox test *p* < 0.0001). ***B***, Climbing assay of the two RNAi lines against *repo* and the rescue of the RNAi 1 by *UAS-repo* as assessed just before death (respectively 7 for RNAi 1, one-way ANOVA, Tukey's multiple-comparisons test *p* = 0.0001 and *p* < 0.0001, respectively, and 12 d for RNAi 2 at 29°C, Mann–Whitney, *n* = 3, *p* = 0.049). ***C***, Western blot analysis of Repo levels after 7 d at 29°C. ***D***, Sixty flies (30 females and 30 males) were used for each genotype. Only *repo* knock-down by the ubiquitous *Actin5cGal4* or the mosaic *dEAAT1-Gal4* achieved a significant detrimental effect on fly lifespan, supporting the composite nature of the effect on lifespan. The specificity of each driver is as follows: *repoGal4* (pan-glial), *alrmGal4* (astrocyte-like glia), *NP2222Gal4* (cortex glia), *moodyGal4* (subperineurial glia), *MZ0709Gal4* (ensheathing glia), *Actin5cGal4* (ubiquitously expressed), *dEAAT1Gal4* (astrocyte-like glia, cortex glia and some subperineurial glia), and *GMRGal4* (eye, used as negative control, not expressed in glial cells). ***p < 0.001.

The regulation of Repo by miR-1 is physiologically relevant during the developmental stages for hemocyte and glial cell specification (Trébuchet et al., 2019). Here, we used miR-1 ectopically as a discovery tool in the adult. Our results suggest that Repo may be specifically required in adult glial cells. It was previously shown that some *repo* alleles lead to neurodegeneration (Xiong and Montell, 1995); however, the use of genetic mutations does not allow us to exclude that neurodegeneration arises as a secondary consequence of developmental abnormalities. The inducible system of expression that we use here allows us instead to specifically investigate functions in adult nervous system maintenance. Adult-specific *repo* RNAi led to dramatic shortening of the fly lifespan (Fig. 2*A*) and progressive loss of motor activity (Fig. 2*B*). Both phenotypes were partially rescued by an exogenous *UAS-repo* (Fig. 2*A*,*B*), which had no effect *per se* (data not shown). This transgene is still sensitive to the RNAi effect, probably explaining the mild degree of phenotypic rescue and of Repo protein expression achieved (Fig. 2*C*). Downregulating *repo* in different subsets of glial cells using other glial Gal4 drivers (astrocyte-like/*alrmGal4*, cortex/*NP2222Gal4*, ensheathing/*MZ0709Gal4*, and subperineural/*moodyGal4* glia) did not markedly affect lifespan (Fig. 2*D*). Only *dEAAT1Gal4* (expressed in astrocyte-like glia, cortex glia, and some subperineurial glia; data not shown) and the ubiquitous *Actin5cGal4* elicited a more robust phenotype in this assay, suggesting that this phenotype results from a combination of factors rather than one single function in a specific glia subset.

**Figure 3.**
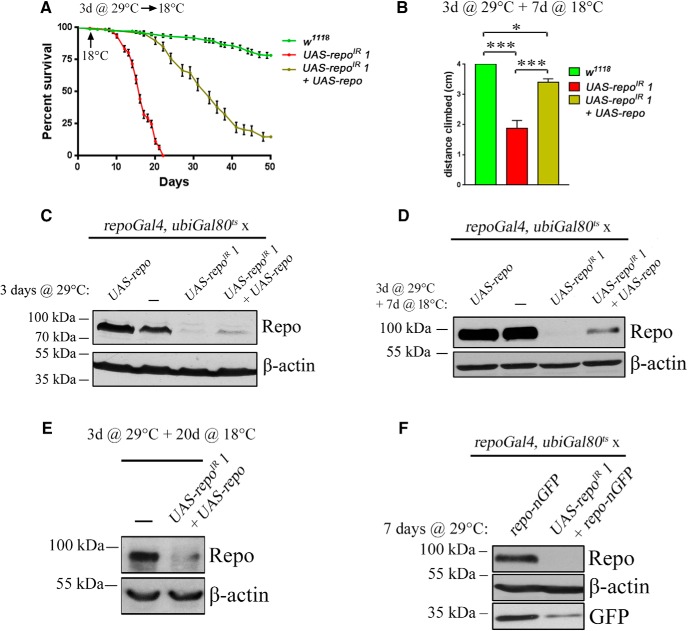
*repo* expression is continuously required. ***A***, Lifespan of *repo*^IR^ *1* flies and their rescue after 3 d of transgene expression at 29°C followed by a block of transgene expression with a transfer at 18°C (indicated by an arrow on the graph). ***B***, Climbing assay of the same genotypes as in ***A*** using the same paradigm. The assay was done after 7 d at 18°C, prior to death of the *repo*^IR^ *1* flies (one-way ANOVA, Fisher's LSD *p* = 0.0001, *p* = 0.0448 and *p* = 0.0007, respectively). ***C***–***E***, Western blot analysis of Repo expression after 3 d at 29°C (***C***). Note the evident overexpression of Repo from an exogenous transgene, which partially counteracts Repo knock-down by the RNAi, after a further 7 d at 18°C (***D***), representing the levels of Repo at the same time point as the climbing assay. Note the absence of Repo overexpression by the *UAS-repo* transgene compared with controls. After 3 d at 29°C and 20 more days spent at 18°C (***E***), the Repo levels in *UAS-repo*^IR^ *1* + *UAS-repo* condition remains very low even after a long period of transgene expression blockage. The driver used was *ubiGal80*^ts^*; repoGal4*. ***F***, Analysis of nGFP protein levels from the *repo-nGFP* transgene after 7 d of *UAS-repo*^IR^ *1* expression. The driver used was *ubiGal80*^ts^*; repoGal4*. *p < 0.05, ***p < 0.001.

We next tested whether *repo* expression is continuously required in the adult fly. At 18°C, *repo* RNAi and an exogenous *UAS-repo* transgene are efficiently repressed by *ubiGal80*^ts^ (data not shown). We have induced *repo* RNAi for the first 3 d of adult life only at 29°C, followed by phenotypic analysis back at 18°C, when transgenes are no longer transcribed. The effects of just 3 d at 29°C on lifespan and motor activity were dramatic (Fig. 3*A*,*B*) and in both cases could be partially rescued by an exogenous *UAS-repo* transgene. At the protein level, 3 d at 29°C led to an efficient knock-down (Fig. 3*C*), but there was no recovery back at 18°C (Fig. 3*D*,*E*). The dramatic effect of a temporary knock-down is therefore likely due to the requirement for the Repo protein to maintain *repo* gene expression. Accordingly, a transgenic *repo-nGFP* was also dramatically reduced by the *repo* RNAi (Fig. 3*F*), confirming the presence of a regulatory sequence within the 4.5 kb *repo* promoter (Lee and Jones, 2005; Flici et al., 2014).

### Expression of genes involved in GABA and glutamate recycling requires Repo in adult glia

The data collected so far indicated a continuous requirement for the Repo protein in adult glia to maintain its own expression, which in turn supports fly lifespan, endogenous locomotor activity, and response behavior to an exogenous mechanical stimulation.

**Figure 4.**
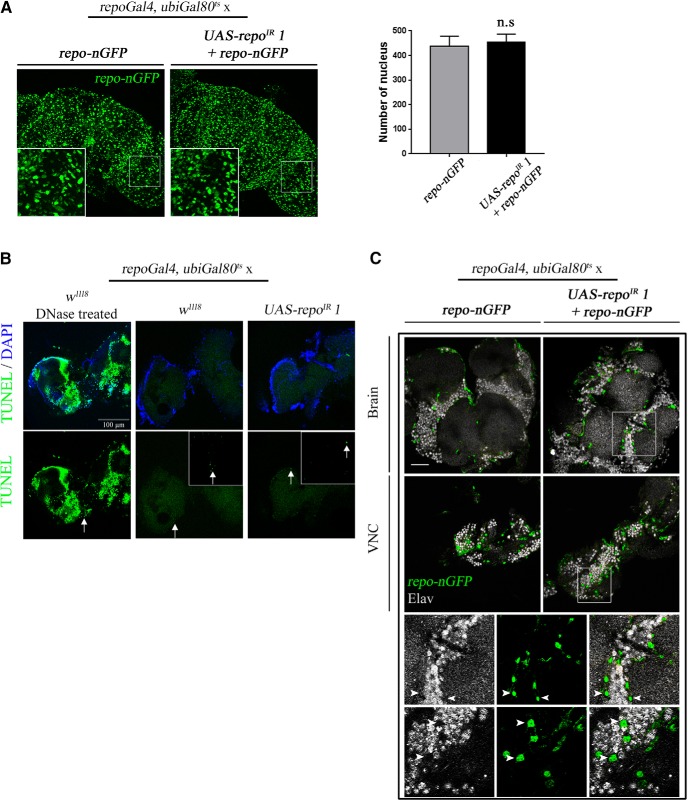
Repo knock-down in the adult does not trigger cell death or change of fate. ***A***, Maximum projections of confocal images of *w*^1118^ and *UAS-repo*^IR^ *1* brains after 7 d at 29°C. Glial cells were labeled in green using the *repo-nGFP* transgene used for previous Western blot (Fig. 3*F*). Quantification was done in the optic lobes of three independent brains for each genotype. No significant difference was observed between control and RNAi conditions. ***B***, TUNEL assay using the same genotypes and the same conditions as previously. A positive control for the assay was done by treating control brains with DNase prior to labeling. No difference could be observed between control and RNAi conditions. See Materials and Methods for detailed protocol. ***C***, Immunostaining of the brain and VNC of *w*^1118^ and *UAS-repo*^IR^ *1* flies after 7 d at 29°C. In green is nGFP from the *repo-nGFP* transgene and in gray is the pan-neuronal marker Elav. The images represent confocal sections at the level of the antennal lobes for the brains and abdominal segment for the Ventral Nerve Cord (VNC). Bottom panels represent higher magnification of the *UAS-repo*^IR^ *1* condition in the brain (top) and in the VNC (bottom). Arrowheads indicate presumptive glial cell nucleus. There was no overlap between GFP and Elav staining in either case. The driver used was *ubiGal80*^ts^*; repoGal4*. Scale bar, 30 μm.

Investigating the possible biological cause for the dramatic phenotype in the *repo*-KD flies, we did not find any evidence for glial cell loss (Fig. 4*A*), apoptotic cell death (Fig. 4*B*), or fate switch of glia to neurons (Fig. 4*C*), all of which take place during development (Trébuchet et al., 2019). We also did not detect gross morphological alterations to the shape of glial cells and of the major nerve fiber tracts (data not shown).

**Figure 5.**
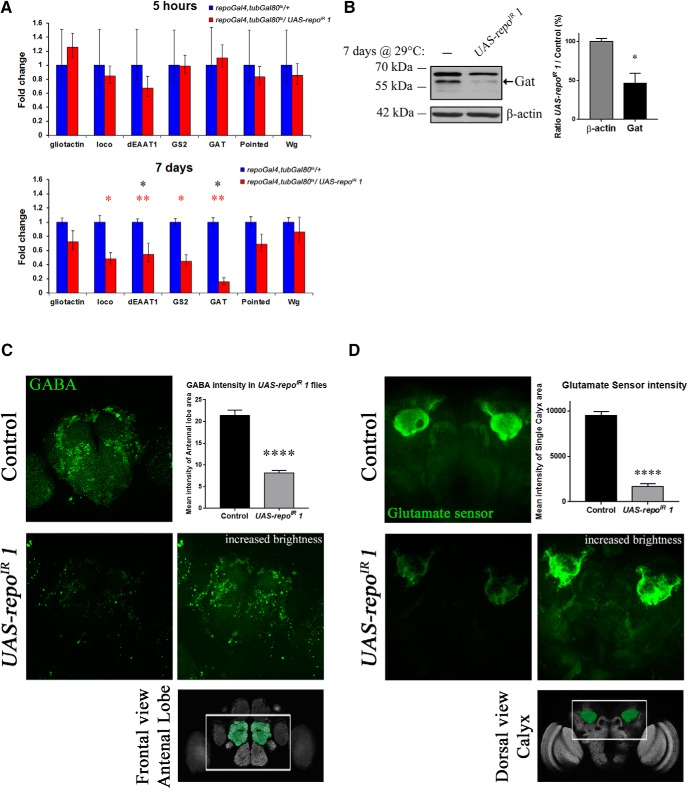
Analysis of Repo targets and effect on GABA and glutamate levels. All aging in this figure was performed at 29°C. ***A***, qPCR analysis of known Repo transcriptional target genes and of *Gat* after 5 h or 7 d at 29°C in *w*^1118^*; tubGal80*^ts^, *repoGal4*/+ (control) and *w*^1118^*; UAS-repo*^IR^ *1*/+*; tubGal80*^ts^, *repoGal4*/+ flies. Each condition was measured with three independent biological replicates. No statistically significant difference was observed between control and the expression of *UAS-repo*^IR^ *1* after 5 h at 29°C (before the RNAi can downregulate *repo*). After 7 d, *loco*, *dEAAT1*, *Gs2*, and *Gat* were significantly downregulated at the mRNA level. Asterisks represent significance before [red, two samples, two-tailed *t* test, *n* = 3 from left to right *p* = 0.0289 (*loco*), *p* = 0.0013 (*dEAAT1*), *p* = 0.0197 (*Gs2*), *p* = 0.0029 (*Gat*)] and after [black, *p* = 0.013 (*dEAAT1*), *p* = 0.0143 (*Gat*)] false discovery rate (FDR) correction. ***B***, Western blot analysis of Gat expression and its quantification. The expected molecular weight of Gat is 57 kDa. The quantification was done with four independent biological replicates. Mann–Whitney, *n* = 4, *p* = 0.0143. ***C***, Immunostaining using a specific anti-GABA antibody (green) of *Drosophila* brains after 7 d at 29°C. The images represent the maximum projection of *z*-stacks of the central brain taken with a 40× oil-immersion objective. The quantification is the average of the pixel intensity given for the area around each antennal lobe for three brains per genotype (unpaired *t* test, two-tailed, *p* < 0.0001). The measurements were done using ImageJ Fiji software. The image of a Repo-RNAi brain with a higher brightness (brightness parameter brought from 255 to 80) shows the presence of the cells and their localization, barely visible under the conditions used for the control brains. A schematic of the brain region observed is highlighted below. ***D***, Fluorescence intensity generated in the adult brain by the glutamate sensor *UAS-iGluSnFRA184S* expressed alone or in combination with *UAS-repo*^IR^ *1* after 7 d at 29°C. The images were taken in the brain area highlighted in the schematic below the pictures. The settings of the microscope were adjusted with the first control brain analyzed and kept unmodified. Quantifications were done using ImageJ Fiji software, drawing a square around each calyx region, measuring the intensity of the brightest slices of the stack, and then averaging (unpaired *t* test, two-tailed, *p* < 0.0001). The genotypes used were as follows: *elav-Gal4; tubGal80*^ts^, *repoGal4/UAS-iGluSnFRA184S* as control and *elav-Gal4; UAS-repo*^IR^ *1*/+*; tubGal80*^ts^, *repoGal4/UAS-iGluSnFRA184S*.

To obtain an indication of the processes that could be disrupted in *repo*-KD flies, we monitored by qPCR the expression of several genes (*Gliotactin*, *loco*, *dEAAT1*, *Gs2*, *pointed*, and *wingless*), recently established as direct targets of Repo (Kerr et al., 2014). *loco* and *Gliotactin* are genes involved in the signaling pathway setting up the fly “blood–brain barrier” (Auld et al., 1995; Schwabe et al., 2005). *pointed* and *wingless* are important for glial specification and development (Klaes et al., 1994; Kerr et al., 2014). In addition, we have examined the expression of the only *Drosophila* glial GABA transporter gene, *Gat*, to complement, together with *dEAAT1* and *Gs2*, the key components of the glutamate/GABA/glutamine cycle (Roth and Draguhn, 2012; Rowley et al., 2012). After 7 d, *loco*, *Gs2*, *Gat*, and *dEAAT1* mRNA levels were significantly downregulated (Fig. 5*A*). Using a specific antibody (Stork et al., 2014), we have confirmed the downregulation of Gat also at the protein level (Fig. 5*B*).

Interestingly, the downregulation of *Gat*, *dEAAT1*, and *Gs2* suggested that the glutamate/GABA/glutamine cycle may be severely compromised in *repo*-KD flies. This cycle in glia has been shown to be of major importance in the maintenance of the glutamate and GABA pool in neurons (Rae et al., 2003). Indeed, antibody staining for the GABA neurotransmitter revealed a striking reduction in GABA levels in neurons (Fig. 5*C*). A similar effect was observed for glutamate as monitored by the iGLUSnFRA184S glutamate sensor (Marvin et al., 2013; Stork et al., 2014). The iGLUSnFRA184S signal was stronger in the calyx and mushroom body regions, which are known to have high levels of glutamate (Daniels et al., 2008; Sinakevitch et al., 2010), and was dramatically decreased in *repo*-KD flies (Fig. 5*D*).

Therefore, *repo* expression in the adult glia is necessary for the maintenance of neurotransmitter homeostasis, likely via the transcriptional regulation of components of the glutamate/GABA/glutamine cycle.

### Gat overexpression in adult glia delays the emergence of motor dysfunction and decreases the sensibility to seizures

**Figure 6.**
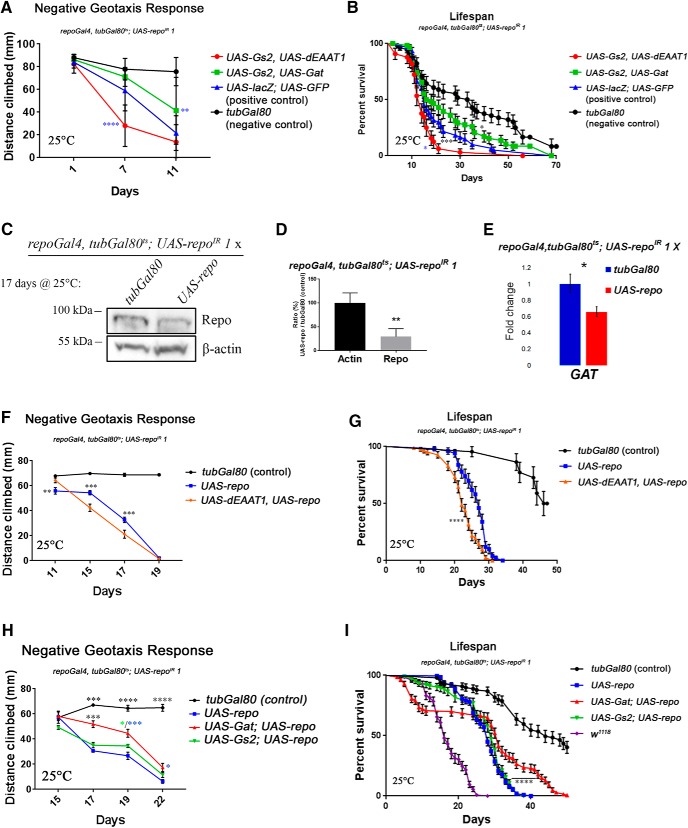
Effect of *Gat*, *Gs2* and *dEAAT1* levels on behavioral and lifespan deficits caused by *repo* knock-down. All rescue experiments in this figure and in the related Figures 7 and 8, were performed at 25°C and genotypes used were *w*^1118^*; UAS-repo*^IR^ *1*/+*; tubGal80*^ts^, *repoGal4/tubGal80* (negative control) // *w*^1118^*; UAS-repo*^IR^ *1/UAS-lacZ; tubGal80*^ts^, *repoGal4/UAS-eGFP* // *w*^1118^*; UAS-repo*^IR^ *1/UAS-Gs2; tubGal80*^ts^, *repoGal4/UAS-dEAAT1* // *w*^1118^*; UAS-repo*^IR^ *1/UAS-Gs2*,*UAS-Gat; tubGal80*^ts^, *repoGal4*/+ // *w*^1118^*; UAS-repo*^IR^ *1/UASeGFP; tubGal80*^ts^, *repoGal4/UAS-repo* // *w*^1118^*; UAS-repo*^IR^ *1/UAS-Gs2; tubGal80*^ts^, *repoGal4/UAS-repo* // *w*^1118^*; UAS-repo*^IR^ *1/UAS-Gat; tubGal80*^ts^, *repoGal4/UAS-repo* **//** *w*^1118^*; UAS-repo*^IR^ *1/UAS-dEAAT1; tubGal80*^ts^, *repoGal4/UAS-repo*. ***A***, Evolution over time of the negative geotaxis response. Ten to 15 flies were assessed three times at different time points at 25°C. Coexpression of *UAS-Gs2* and *UAS-Gat* delays the decrease in climbing ability over time compared with the positive control, whereas coexpression of *UAS-Gs2* and *UAS-dEAAT1* transiently aggravates the phenotype. Blue asterisks indicate significance compared with the positive control (two-way ANOVA, Tukey's multiple-comparisons test *p* < 0.0001 and *p* = 0.0011, respectively). ***B***, Fly lifespan was assessed with control flies carrying the *tubGal80* transgene (not temperature sensitive) to block the Gal4 activity. The positive control flies expressing control proteins in a *repo*-KD background display a significantly shorter lifespan compared with the negative control. Coexpression of *UAS-Gs2* and *UAS-dEAAT1* further shortens the lifespans of *repo*-KD flies, whereas coexpression of *UAS-Gs2* and *UAS-Gat* displays a trend toward rescue but no significant difference. Black asterisks indicate significance compared with the negative control; blue asterisks indicate significance against the positive control (log–rank Mantel–Cox test *p* = 0.0207, *p* = 0.0001 and *p* = 0.0134, respectively). ***C***, Western blot showing the downregulation of Repo protein in rescued-*repo*-KD compared with control flies. Actin is used as a loading control. ***D***, Quantification of Repo protein expression. Mann–Whitney, *n* = 6, *p* = 0.0022. ***E***, UPL qPCR assay for *Gat* (as performed in Fig. 5*A*). Values are normalized using *eIF4A* as control. Unpaired *t* test, two-tailed, *n* = 3 *p* = 0.024. ***F***, Evolution over time of the negative geotaxis response as in ***A***. Coexpression of *UAS-dEAAT1* and *UAS-repo* accelerates the decrease in climbing ability over time. Black asterisks indicate significance compared with the negative control (two-way ANOVA, Tukey's multiple-comparisons test, *p* = 0.0068, *p* = 0.0001, and *p* = 0.0003, respectively). ***G***, Fly lifespan was assessed as in ***B***. Similar to the climbing assay, the coexpression of *UAS-dEAAT1* beside *UAS-repo* does not rescue and rather aggravates the lifespan phenotype. Asterisks indicate significance compared with *UAS-repo* alone (log–rank Mantel–Cox test *p* = 0.000010383935). ***H***, Evolution over time of the negative geotaxis response as in ***A***. The flies coexpressing *UAS-Gat* and *UAS-repo* display a significantly improved climbing abilities compared with *UAS-repo* alone or *UAS-repo* and *UAS-Gs2*. Black asterisks indicate significance in genotype/time interactions compared with *UAS-repo* alone in two-way ANOVA (Tukey's multiple-comparisons test *p* < 0.0001, *p* = 0.0009, *p* < 0.0001, and *p* < 0.0001, respectively). Colored asterisks indicate significance between the different genotypes and *UAS-repo* alone at a specific time point (main genotype factor in two-way ANOVA, Tukey's multiple-comparisons test *p* = 0.0408, *p* = 0.0001 and *p* = 0.0207, respectively). ***I***, Conditions of the lifespan similar to ***G***, but with the addition *repo*-KD flies as a positive control genotype (*w*^1118^*; UAS-repo*^IR^ *1*/+*; tubGal80*^ts^, *repoGal4*/+). The coexpression of *UAS-Gs2* does not affect the lifespan, whereas *UAS-Gat* mildly but significantly increases the lifespan of 70% of the flies. Surprisingly, ∼30% of the flies in this condition die within 10 d, whereas 25–30% of the population have a strong increase in lifespan. Asterisks indicate significance compared with *UAS-repo* alone (log–rank Mantel–Cox test *p* = 0.000000018838).

Rescue experiments to analyze the causal role of the downregulation of genes controlling the glutamate/GABA/glutamine cycle for the *repo*-KD phenotypes were performed at 25°C instead of 29°C to allow slower progression of defects. As negative control for the phenotypes analyzed, we have used *tubGal80* to inhibit expression of all transgenes while maintaining all common genomic elements than all other stocks analyzed (*repoGal4*, *tubGal80*^ts^, and *UAS-repo*^IR^ *1*). The positive controls were *repo*-KD flies expressing control proteins LacZ and GFP. These flies were not included in all assays because they become immobile or die too early for meaningful comparison at the late stages. Gal4/UAS mediated overexpression of only one of the genes tested (*Gat*, *Gs2*, and *dEAAT1*) was unable to rescue either motor defects or lifespan in *repo*-KD flies (data not shown). However, a mild but significant rescue was detected upon *Gs2* and *Gat*, but not *Gs2* and *dEAAT1* co-overexpression in climbing assays (Fig. 6*A*). The coexpression of *Gs2* and *dEAAT1* was rather transiently detrimental in this assay and in lifespan analysis (Fig. 6*B*). At the level of lifespan, the coexpression of *Gs2* and *Gat* only displayed a trend toward rescue, but was not significantly different from the positive control (Fig. 6*B*). To determine the individual contribution of single genes involved in the glutamate/GABA/glutamine cycle, we expressed them separately in synergy with an exogenous *repo* transgene in *repoGal4*, *ubiGal80*^ts^>*UAS-repo*^IR^, *UAS-repo* (hereafter rescued-*repo*-KD) flies. When aged at 25°C, the rescued-*repo*-KD flies still display a downregulation of Repo to ∼1/3 of control levels (Fig. 6*C*,*D*) and a milder but significant downregulation of *Gat* expression at ∼2/3 of control levels (Fig. 6*E*). Therefore, this represents an ideal background in which to assess the effect of exogenous expression of each of the three components of the glutamate/GABA/glutamine cycle analyzed here. Coexpression of *UAS-dEAAT1* in rescued-*repo*-KD flies rather resulted in a detrimental effect both in the negative geotaxis climbing assay (Fig. 6*F*) and in lifespan (Fig. 6*G*) similar to what observed in combination with Gs2 (Fig. 6*A*,*B*). In contrast, coexpression of *UAS-Gat* significantly improved the climbing ability in rescued-*repo*-KD flies (Fig. 6*H*). At the level of lifespan, a mild but significant rescue was detected (Fig. 6*I*), but resulted from a consistent biphasic effect. The reasons for the biphasic lifespan of rescued-*repo*-KD flies coexpressing *UAS-Gat* are unknown and were not present when *Gat* was expressed alone or in combination with *Gs2* (Fig. 6*B*).

**Figure 7.**
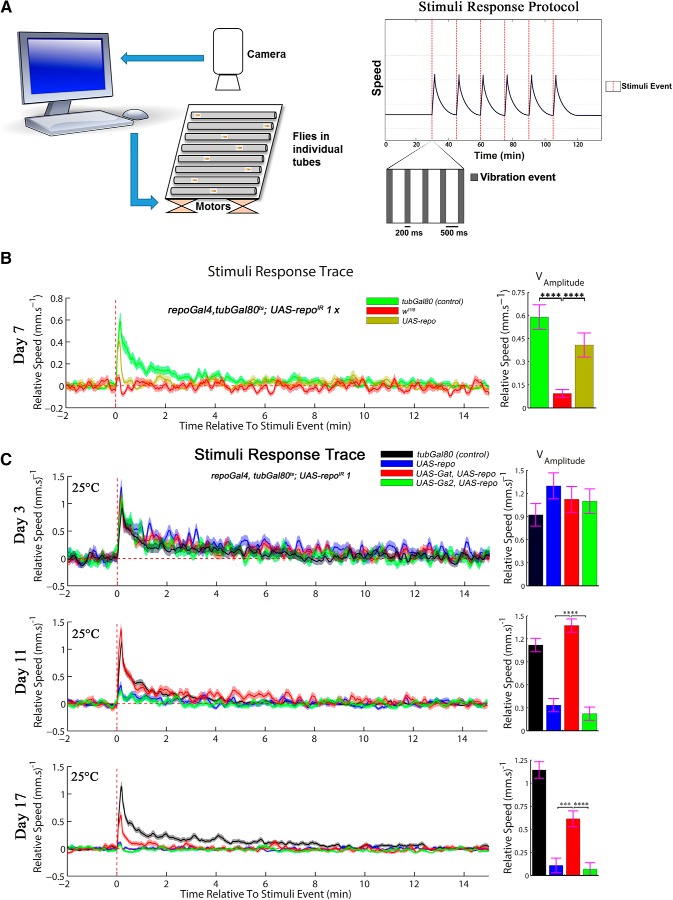
Novel DART paradigm for sophisticated analysis dissects the role of *Gat* and *Gs2* in exogenous motor activity. ***A***, Schematic summary of the hardware and behavioral protocol used for the DART stimuli response. See Material and Methods for details of the setup. ***B***, Behavioral analysis of the *repo*-KD flies using an automated setup recording the response to a stimulus at a single fly level. After 7 d at 29°C, the *repo*-KD flies fail to respond to given stimuli (provided by shaft-less motors placed underneath the behavioral platform) and the effect is significantly rescued by the coexpression of a *UAS-repo* transgene (one-way ANOVA, Dunnett's multiple-comparisons test *p* < 0.0001 for both). Twenty flies were assessed per genotype. The genotypes were as follows: *w*^1118^*; UAS-repo*^IR^ *1; repoGal4*, *tubGal80*^ts^ crossed with either: *w*^1118^*; tubGal80* (negative control for the phenotype), *w*^1118^ *or w*^1118^*; UAS-repo*. ***C***, Behavioral analysis of stimuli response (exogenous motor activity) of the same genotypes as in Figure 6*E*, at 3, 11, and 17 d at 25°C. Twenty flies were analyzed for each genotype at each time point. The histograms represent the amplitude of the peaks. The coexpression of *UAS-Gat* and *UAS-repo* significantly improved the flies' performance compared with *UAS-repo* alone or *UAS-repo* and *UAS-Gs2* (one-way ANOVA, Dunnett's multiple-comparisons test *p* < 0.0001 for both at day 11 and *p* = 0.0006 and *p* < 0.0001 at day 17.). ***p < 0.001, ****p < 0.0001.

Given the pleiotropy of lifespan effects, we reasoned that a different assay was necessary to effectively determine all components involved in the regulation of the glutamate/GABA/glutamine cycle by Repo. Therefore, we focused our analysis on specific behavioral deficits in the *repoGal4*, *ubiGal80*^ts^>*UAS-repo*^IR^ flies (hereafter *repo*-KD). For refined and unbiased investigations, we established a novel DART paradigm (Faville et al., 2015). This allows automated video-assisted motion tracking and stimuli response to mechanical shock to investigate endogenous and exogenous locomotor activity (Fig. 7*A*; see Materials and Methods for detailed protocol). After 7 d at 29°C, whereas control flies (that carry all relevant transgenes, however repressed by *tubGal80*) respond to the series of vibrations by a sudden transient increase in speed (represented by a peak in relative speed), the *repo*-KD flies did not respond to the stimuli provided (Fig. 7*B*). This defect was also partially but significantly rescued by the expression of an exogenous *UAS-repo*. Therefore, our novel DART setup is capable of robust and sensitive analysis of motor behavior.

Using our DART setup, we analyzed the effect of *UAS-Gat* in more detail. Indeed, whereas all tested genotypes are indistinguishable after 3 d at 25°C, after 11 d at 25°C (a stage when climbing defects are not yet observed in rescued-*repo*-KD flies), this setup is able to detect a dramatic reduction in the response to the given stimuli. At this stage, coexpression of *UAS-Gat* significantly improves the amplitude of response to the level of the control flies, in which all transgenes are silenced and this improvement is still present after 17 d at 25°C (Fig. 7*C*).

**Figure 8.**
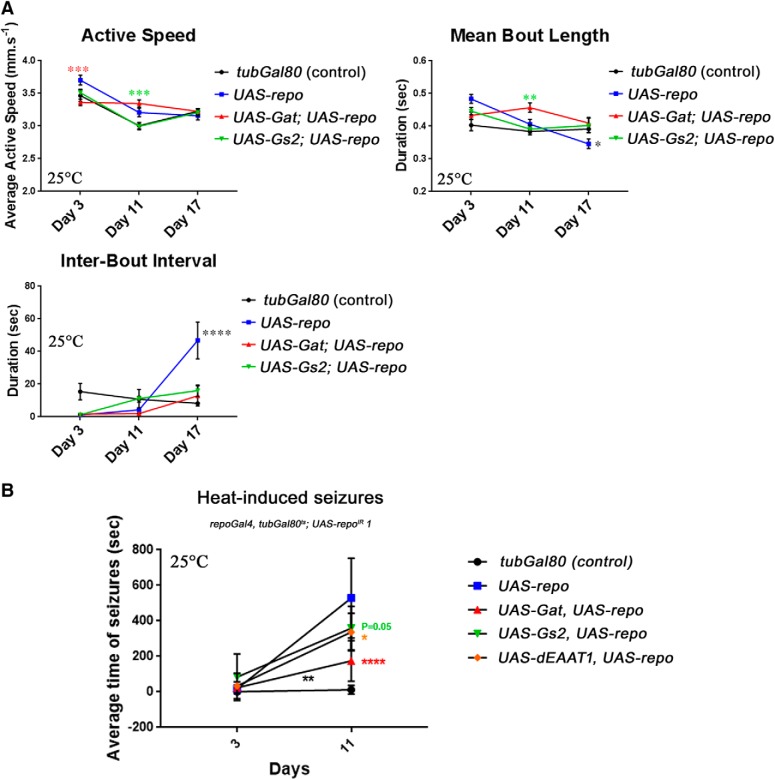
Effect of Gs2 and Gat on endogenous motor activity and recovery from seizures. ***A***, Constant tracking of the flies allows the analysis of the endogenously generated motor activity represented here by the three functions active speed, mean bout length, and interbout interval (see Materials and Methods). The flies and data used are the same as in Figure 7*C*. Although the active speed does not seem to be strongly affected, the flies expressing only *UAS-repo* beside *UAS-repo*^IR^ *1* have a significant reduction in mean bout length after 17 d and a strong increase of the interbout interval also after 17 d. These changes are not present when either *UAS-Gat* or *UAS-Gs2* is coexpressed. For simplicity, statistical comparisons represented are between *UAS-repo* vs *UAS-Gat* + *UAS-repo* and *UAS-repo* vs *UAS-Gs2* + *UAS-repo* only (two-way ANOVA, Tukey's multiple-comparisons test *p* = 0.0004, *p* = 0.0002, *p* = 0.0077, *p* = 0.0300, and *p* < 0.0001, respectively). ***B***, Time to recovery analysis of epileptic-like features at 3 and 11 d at 25°C. Ten flies were analyzed for each genotype at each time point, repeated five times independently, and averaged. *UAS-repo* and *UAS-Gat* co-overexpression significantly rescued the seizure phenotype due to *repo* knock-down. Black asterisks indicate significance in genotype/time interactions compared with *UAS-repo* alone (two-way ANOVA, Dunnett's multiple-comparisons test *p* = 0.0029). Colored asterisks indicate significance between the different genotypes and *UAS-repo* alone at a specific time point (two-way ANOVA, Dunnett's multiple-comparisons test *p* = 0.0545, *p* = 0.0248, and *p* < 0.0001, respectively).

Surprisingly, the defects in stimuli response by rescued-*repo*-KD flies do not arise because the flies are slower when active. Indeed, their speed while active was comparable to control unaffected flies at days 11 and 17 and, if anything, even faster at day 3 (Fig. 8*A*). Consistently, the rescue operated by *UAS-Gat* was not due to an increase of this parameter.

Interestingly, when analyzing the overall activity of these flies for the whole 2 h of the assay paradigm, not limited therefore to the sole response to stimulus and more representative of endogenously generated motor activity, it is apparent that the rescued-*repo*-KD flies display a tendency to decrease the length of their actions (mean bout length) while at the same time increasing the pauses in between actions (interbout interval), with both parameters being significantly different from the other genotypes at day 17 (Fig. 8*A*). Therefore, in addition to a dramatic decrease in response capability, these flies display also a trend toward endogenous inactivity that was highly significant after 17 d and rescued by *UAS-Gat*.

Interestingly, coexpression of *UAS-Gs2* improved the endogenous mode of activity, with flies coexpressing *Gs2* and *repo* displaying similar mean bout length and interbout intervals to control flies and flies rescued by *repo-Gat* coexpression (Fig. 8*A*). However, compared with *UAS-Gat*, coexpression of *UAS-Gs2* failed to provide any significant rescue in the response-triggered behavior in the climbing assay and in lifespan (Fig. 6*H*,*I*), as well as in the DART setup (Fig. 7*C*), indicating a more limited effect for *Gs2*.

In mammals, the orthologes of *dEAAT1*, *Gs2*, and *Gat* are involved in sensitivity to seizures (Pirttimaki et al., 2013; Steinhäuser et al., 2016; Boison and Steinhäuser, 2018) and, recently, a role of *dEAAT1* has been reported also in *Drosophila* (Cho et al., 2018). To test the effect on this important glial function for the same genotypes, we measured the time to recovery using a well established procedure to trigger seizures in flies (Sun et al., 2012). Similar to stimuli–response, we did not observe any significant differences in 3 d adult flies between all the genotypes assessed (Fig. 8*B*). However, after 11 d, the rescued-*repo*-KD flies developed very long-lasting seizures of up to 25–30 min, with an average time to recovery of 9 min, compared with control flies, which have average seizures of 12 s, similar to the early time point (Fig. 8*B*). Interestingly, the coexpression of *UAS-Gat* improved this defect, whereas *UAS-Gs2* and *UAS-dEAAT1* did not significantly modify its progression with aging. When considering only the time point at 11 d, *UAS-Gat* coexpression decreased significantly the time to recovery to 3 min, whereas *UAS-Gs2* or *UAS-dEAAT1* only mildly affected the phenotype (Fig. 8*B*).

Together, these data suggest that the downregulated expression of *Gat* is, at least partially, specifically responsible for the strong behavioral defects due to the loss of Repo, likely through the alterations of the glutamate/GABA/glutamine cycle and the resulting dramatic reduction in GABA and glutamate levels in the brain.

## Discussion

### Repo is specifically needed throughout *Drosophila* adult life

Repo, the key glia determinant in *Drosophila*, has been mostly studied in embryonic and larval stages, where it is involved in terminal glial differentiation and migration, activating the expression of several genes, some specific to glial cells. Even though constantly expressed in almost all glial cells throughout life and a recognized association of some alleles with neurodegeneration (Xiong and Montell, 1995), its specific role in the adult has largely been neglected, with the exception of a recent study concerning its role in learning and memory (Matsuno et al., 2015).

Here, starting from an miRNA screen, we identified an additional form of regulation of Repo levels through miR-1, having demonstrated in a separate study that this regulation is physiologically relevant in hemocyte development (Trébuchet et al., 2019). Here, we use it ectopically as a discovery tool that validates the ability of miRNA-based screens to inform on the specific relationship with one target gene, even via ectopic miRNA expression.

We show next that *repo* is continuously required to maintain a viable and fully functional organism because, once its expression is abolished or strongly decreased, *repo* does not manage to reinstate its initial levels. Indeed, a 3 d downregulation was enough to irreversibly stop *repo* expression. Therefore, Repo is continuously required to maintain itself and, thus, a functional nervous system. A model for the molecular basis for this always-ON/always-OFF autoregulation has been proposed in development (Lee and Jones, 2005); however, the simultaneous effects on glial cell fate and number made it difficult to distinguish loss of transcription, from loss of cells. Because such confounding effects are not present in the adult, we provide here unequivocal evidence for *repo* autoregulation.

### Transcriptional regulation of neurotransmitter recycling in adult glial cells affects *Drosophila* motor activity and recovery from seizures

During glial differentiation, *repo* triggers the expression of genes such as *dEAAT1* and *Gs2*, both involved in neurotransmitter recycling. Moreover, downregulation of the glial glutamate transporter *dEAAT1* and GABA transporter *Gat* during larval stages triggers dramatic motor defects (Rival et al., 2004; Muthukumar et al., 2014). Here, we show that the transcription of two established direct Repo target genes, *dEAAT1* and *Gs2* (Kerr et al., 2014), require Repo also during adulthood. Furthermore, we demonstrate for the first time that Repo is also necessary to maintain the expression of the only *Drosophila* glial GABA transporter, *Gat*, both at the mRNA and protein levels. However, in the case of *Gat*, it is likely that its levels are regulated by Repo via an indirect mechanism. No Repo binding sites are present in its promoter and gene sequence, in contrast to *dEAAT1* and *Gs2*. Additional unidentified factors may therefore mediate the effect of Repo on *Gat*, possibly also through the effect on neurotransmitters levels. Although additional factors may mediate glial subtype specificity, considering that not only astrocytes but also ensheathing glia regulate glutamate homeostasis in *Drosophila* (Otto et al., 2018), our data strongly suggest that *repo* is a strictly required top-controller of the highly conserved glutamate/GABA/glutamine cycle in the *Drosophila* adult nervous system, governing directly and indirectly the expression of key components.

Consistent with these data, the glial expression of *UAS-Gat* significantly enhanced the partial rescue by an exogenous *UAS-repo* of the motor defects and epileptic-like features caused by knock-down of the endogenous *repo*. Althouh neither *dEAAT1* nor *Gs2* coexpression could rescue all these defects, our genetic setup combining different tools cannot rule out a possible contribution of these two genes to the control of the glutamate/GABA/glutamine cycle orchestrated by Repo in glia.

To reach these conclusions, we have used a combination of approaches that, from a coarse lifespan analysis, progressed through negative geotaxis responses and to the development of a sophisticated behavioral monitoring system. The key advantage of this approach is that it has allowed us to separate progressively the effect of gene level dysregulation at different levels of refinement, being able to detect events not evident through lifespan analysis or detected much earlier than in simple negative geotaxis assays. This novel behavioral paradigm can therefore be applied successfully to handle more subtle effects in the increasingly popular *Drosophila* models for neurological disorders.

The precise role of glial GABA transporters in glia is still unclear, considering their potential of clearing GABA from the synapse, but also releasing it extrasynaptically to the postsynaptic neuron as tonic inhibition (Héja et al., 2012). Moreover, even though the glutamate/GABA/glutamine cycle has been extensively studied in mammalian models (Héja et al., 2012; Shameem and Patel, 2012; El-Khoury et al., 2014; Pehrson and Sanchez, 2015; Zheng et al., 2016), the transcriptional regulation of its key genes has been poorly addressed despite its importance in epilepsy, Huntington's disease, and psychiatric disorders (Eid et al., 2013; Huyghe et al., 2014; Cvetanovic et al., 2015; Karki et al., 2015; Boison and Steinhäuser, 2018). This resonates with a wider gap in knowledge of the glial cell functions that contribute to the regulation of neuronal activity via recycling of neurotransmitters. The implication of glia in disease conditions such as epilepsy or psychiatric disorders, where neurotransmitter balance is known to be impaired, has only recently been investigated, particularly for the astrocytic GABA transporters. Indeed, *Gat* levels in *Drosophila* astrocytes are modulated throughout development via metabotropic GABA receptor signaling and *Gat* regulation can modulate seizure activity (Muthukumar et al., 2014). In addition, TRP channels act on the astrocyte-specific mammalian ortholog GAT-3, regulating its membrane trafficking and/or recycling rate through calcium signaling (Shigetomi et al., 2011). However, nothing is known on transcriptional regulation of GABA transporter genes in mammals. Given their role in epilepsy (Pirttimaki et al., 2013; Schousboe and Madsen, 2017), it is essential to better understand the full extent of their regulation at all levels. The discovery of the importance of *repo* in maintaining the neurotransmitter balance in the glutamate/GABA/glutamine cycle point toward the importance of understanding the transcriptional regulation, and could provide a useful and tractable model to unravel the glial contribution in human disease with neurotransmitter imbalance.
