# Supplementary material for: Transcriptional Regulation of the Glutamate/GABA/Glutamine Cycle in Adult Glia Controls Motor Activity and Seizures in Drosophila
Source: J Neurosci. 2019 Jul 3;39(27):5269–83. doi: 10.1523/JNEUROSCI.1833-18.2019 (PMC6607755; doi:10.1523/JNEUROSCI.1833-18.2019)
Supplement: Figure 1-1 [file zns999191741so1.docx]

| **Extended Data Figure 1-1** | | | | | |
| --- | --- | --- | --- | --- | --- |
|  |  |  |  |  |  |
|  | **miRNAs** | **Lines** | **Median survival** | ***χi²*** | **p-values** |
|  | **1** | **7091** | **14** | **88.46** | **1E-12** |
|  |  | **7090** | **14** | **0.015** | **0.90154729** |
|  | **2** | **7041** | **27** | **1.79** | **0.18059999** |
|  |  | **7042** | **24** | **0.29** | **0.58895068** |
|  | **2b** | **7201** | **27** | **0.2** | **0.65572743** |
|  |  | **7202** | **36** | **48.8** | **3E-12** |
|  | **2.5** | **7028** | **28** | **2.7** | **0.10064976** |
|  |  | **7030** | **28** | **0.51** | **0.47448112** |
|  | **3** | **7283** | **15** | **66.52** | **1E-12** |
|  |  | **7281** | **18** | **43.49** | **4.3E-11** |
|  | **4** | **7284** | **39** | **78.39** | **1E-12** |
|  |  | **7286** | **21** | **20.65** | **5.5062E-06** |
|  | **5** | **7270** | **36** | **41.79** | **1.01E-10** |
|  |  | **7271** | **30** | **11.15** | **0.00083816** |
|  | **6** | **7032** | **28** | **0.13** | **0.71834897** |
|  |  | **7033** | **33** | **20.29** | **6.6556E-06** |
|  | **7** | **7039** | **28** | **13.5** | **0.00023868** |
|  |  | **7204** | **33** | **27.84** | **1.3164E-07** |
|  | **Let-7** | **7239** | **30** | **11.92** | **0.00055471** |
|  |  | **7240** | **33** | **19.65** | **9.2789E-06** |
|  |  | **7247** | **18** | **49.17** | **2E-12** |
|  | **8** | **7192** | **27** | **0.1** | **0.75276329** |
|  |  | **7193** | **24** | **23.64** | **1.1613E-06** |
|  | **9a** | **7093** | **11** | **98.69** | **1E-12** |
|  |  | **7094** | **11** | **90.27** | **1E-12** |
|  | **9b** | **7742** | **9** | **106.57** | **1E-12** |
|  |  | **7743** | **9** | **106.46** | **1E-12** |
|  | **9c** | **7179** | **6** | **106.03** | **1E-12** |
|  |  | **7747** | **9** | **28.93** | **7.5044E-08** |
|  | **mirtron-9** | **823** | **31** | **8.21** | **0.00415845** |
|  |  | **822** | **29.5** | **0.05** | **0.82967891** |
|  | **10** | **7398** | **26** | **9.45** | **0.00211216** |
|  |  | **7400** | **26** | **2.2** | **0.13793887** |
|  | **11** | **7206** | **27** | **0.27** | **0.60464602** |
|  |  | **7208** | **30** | **6.35** | **0.01171296** |
|  | **12** | **7253** | **21** | **36.26** | **1.73E-09** |
|  |  | **7255** | **27** | **8.17** | **0.00424836** |
|  | **14** | **7197** | **30** | **6.54** | **0.0105297** |
|  |  | **7199** | **33** | **30.35** | **3.6002E-08** |
|  | **31** | **7099** | **30** | **0.1** | **0.75534876** |
|  |  | **7100** | **25.5** | **5** | **0.02528433** |
|  |  | **7104** | **27** | **0.07** | **0.7957011** |
|  | **31b** | **7319** | **33** | **10.29** | **0.0013349** |
|  |  | **7320** | **35** | **24.29** | **8.3032E-07** |
|  | **33** | **7171** | **28.5** | **0.48** | **0.48901892** |
|  |  | **7173** | **30** | **8.23** | **0.00412341** |
|  | **34** | **7251** | **18** | **64.49** | **1E-12** |
|  |  | **7252** | **12** | **93.17** | **1E-12** |
|  |  | **7045** | **22** | **27.36** | **1.6883E-07** |
|  | **79** | **7101** | **16** | **74.55** | **1E-12** |
|  |  | **7102** | **16** | **76.78** | **1E-12** |
|  | **87** | **7103** | **24.5** | **18.66** | **1.5655E-05** |
|  | **92a** | **7111** | **11** | **90.36** | **1E-12** |
|  |  | **7112** | **11** | **100.85** | **1E-12** |
|  | **92b** | **7287** | **18** | **56.6** | **1E-12** |
|  | **100** | **7237** | **36** | **39.66** | **3.02E-10** |
|  |  | **7238** | **39** | **76.99** | **1E-12** |
|  | **124** | **7108** | **12** | **87.98** | **1E-12** |
|  |  | **7106** | **16** | **77.16** | **1E-12** |
|  | **125** | **835** | **31** | **12.88** | **0.0003325** |
|  |  | **834** | **36** | **34.19** | **4.998E-09** |
|  | **133** | **7291** | **21** | **51.6** | **1E-12** |
|  |  | **7292** | **18** | **44.44** | **2.6E-11** |
|  | **137** | **7068** | **14** | **85.48** | **1E-12** |
|  | **184** | **7295** | **15** | **33.27** | **8.033E-09** |
|  |  | **7294** | **21** | **21.54** | **3.4696E-06** |
|  | **190** | **7787** | **34** | **28.03** | **1.1958E-07** |
|  | **193** | **7478** | **17** | **37.13** | **1.107E-09** |
|  |  | **7476** | **17** | **57.93** | **1E-12** |
|  | **210** | **7273** | **21** | **12.47** | **0.00041435** |
|  |  | **7278** | **30** | **4.64** | **0.03118434** |
|  | **219** | **7109** | **27** | **4.31** | **0.03783169** |
|  | **252** | **7190** | **36** | **37.75** | **8.06E-10** |
|  |  | **7191** | **33** | **27.02** | **2.0133E-07** |
|  | **263a** | **7113** | **30** | **8.88** | **0.00288785** |
|  |  | **7115** | **25.5** | **2.68** | **0.10142083** |
|  | **263b** | **7480** | **27** | **3.91** | **0.04806325** |
|  |  | **7484** | **27** | **7.22** | **0.0072113** |
|  | **274** | **7119** | **21** | **28.15** | **1.1226E-07** |
|  |  | **7117** | **24** | **17.13** | **3.4834E-05** |
|  | **275** | **7296** | **27** | **8.14** | **0.00432396** |
|  |  | **7297** | **31** | **10.11** | **0.00147316** |
|  | **276a** | **7121** | **30** | **7.81** | **0.00518572** |
|  | **276b** | **7265** | **21** | **45.46** | **1.6E-11** |
|  |  | **7266** | **21** | **37.41** | **9.57E-10** |
|  | **277** | **7247** | **18** | **49.17** | **2E-12** |
|  | **278** | **7124** | **16** | **83.5** | **1E-12** |
|  |  | **7125** | **13** | **71.76** | **1E-12** |
|  | **279** | **7127** | **24** | **27** | **2.0303E-07** |
|  | **280** | **817** | **37** | **42.94** | **5.6E-11** |
|  |  | **7175** | **28** | **0.36** | **0.54760209** |
|  | **281** | **7486** | **28.5** | **10.2** | **0.00140612** |
|  |  | **7487** | **31** | **2.25** | **0.13331418** |
|  |  | **7491** | **19** | **33.88** | **5.853E-09** |
|  |  | **7494** | **35** | **21.04** | **4.4957E-06** |
|  |  | **7495** | **29** | **0.66** | **0.41643639** |
|  |  | **7499** | **28** | **3.62** | **0.05710792** |
|  | **282** | **7130** | **26** | **8.33** | **0.00388451** |
|  |  | **7131** | **31** | **9.87** | **0.00167931** |
|  | **283** | **7260** | **37.5** | **46.59** | **9E-12** |
|  | **284** | **7500** | **26** | **0.04** | **0.84560188** |
|  |  | **7502** | **28.5** | **0.05** | **0.83013561** |
|  | **285** | **7132** | **24** | **1.04** | **0.30672689** |
|  |  | **7135** | **25** | **0.71** | **0.39937673** |
|  | **286** | **7300** | **35** | **26.89** | **2.1504E-07** |
|  |  | **7301** | **33** | **13.09** | **0.00029729** |
|  | **287** | **7136** | **26** | **4.51** | **0.0337926** |
|  | **288** | **7302** | **30** | **8.16** | **0.00428997** |
|  |  | **7303** | **30** | **7.82** | **0.00517646** |
|  | **303** | **7141** | **26** | **1.22** | **0.26924035** |
|  |  | **7143** | **26** | **0.07** | **0.78717041** |
|  | **304** | **7255.5** | **36** | **64.7** | **1E-12** |
|  |  | **7256** | **33** | **14.36** | **0.00015068** |
|  | **305** | **7305** | **27** | **0.003** | **0.95242357** |
|  |  | **7306** | **21** | **0.17** | **0.67838341** |
|  | **307** | **7144** | **29** | **0.47** | **0.4914377** |
|  |  | **7145** | **27** | **1.43** | **0.23237207** |
|  | **308** | **7146** | **32** | **16.38** | **5.1733E-05** |
|  |  | **7150** | **38** | **41.04** | **1.49E-10** |
|  | **309** | **7308** | **27** | **0.19** | **0.66600005** |
|  |  | **7309** | **33** | **19.82** | **8.5207E-06** |
|  | **310** | **7310** | **11** | **98.81** | **1E-12** |
|  |  | **7312** | **9** | **97.43** | **1E-12** |
|  |  | **7151** | **6** | **111.21** | **1E-12** |
|  | **312** | **7313** | **22.5** | **21.59** | **3.3761E-06** |
|  |  | **7314** | **15** | **44.18** | **3E-11** |
|  | **313** | **7316** | **18** | **62.96** | **1E-12** |
|  |  | **7317** | **21** | **38.46** | **5.58E-10** |
|  | **314** | **7152** | **29.5** | **10.91** | **0.00095524** |
|  |  | **7153** | **31** | **18.11** | **2.0801E-05** |
|  | **315** | **7158** | **21** | **38.62** | **5.15E-10** |
|  |  | **7156** | **24** | **10.56** | **0.00115586** |
|  | **316** | **7159** | **32** | **0.8** | **0.37189383** |
|  |  | **7160** | **27** | **4.59** | **0.03219133** |
|  | **317** | **7163** | **27** | **5.3** | **0.02127374** |
|  |  | **7165** | **33** | **20.14** | **7.2047E-06** |
|  | **318** | **7168** | **21** | **44.17** | **3E-11** |
|  |  | **7169** | **25.5** | **8.6** | **0.00336719** |
|  | **375** | **7792** | **15** | **78.5** | **1E-12** |
|  | **927** | **7504** | **30** | **0.93** | **0.3345061** |
|  |  | **7505** | **32** | **13.58** | **0.00022873** |
|  | **929** | **827** | **31** | **17.1** | **3.549E-05** |
|  |  | **829** | **28.5** | **4.68** | **0.03045992** |
|  | **932** | **7507** | **28** | **3.55** | **0.05961146** |
|  |  | **7508** | **20** | **48.19** | **4E-12** |
|  | **954** | **7511** | **27** | **0.09** | **0.76262793** |
|  |  | **7512** | **27** | **0.42** | **0.51718132** |
|  | **955** | **7515** | **30** | **3.98** | **0.04607133** |
|  |  | **7517** | **30** | **0.69** | **0.40775384** |
|  | **956** | **809** | **31** | **9.44** | **0.00212625** |
|  |  | **810** | **27** | **10.01** | **0.00155484** |
|  | **957** | **7519** | **29** | **0.003** | **0.95430859** |
|  |  | **7520** | **33** | **15.59** | **7.863E-05** |
|  | **958** | **7523** | **22** | **37.11** | **1.116E-09** |
|  |  | **7526** | **25.5** | **13.73** | **0.00021087** |
|  | **959** | **7528** | **37** | **55.37** | **1E-12** |
|  |  | **7529** | **24** | **20.61** | **5.6332E-06** |
|  | **960** | **7544** | **31** | **0.57** | **0.4493973** |
|  |  | **7545** | **26** | **5.55** | **0.01848196** |
|  |  | **7549** | **31** | **10.32** | **0.00131879** |
|  |  | **7550** | **27** | **1.79** | **0.18097355** |
|  | **962** | **7557** | **31** | **6.35** | **0.01174429** |
|  |  | **7558** | **31** | **10.71** | **0.00106548** |
|  | **963** | **7560** | **38** | **58.82** | **1E-12** |
|  |  | **7561** | **31** | **11.66** | **0.00063874** |
|  | **964** | **7568** | **28** | **0.0004** | **0.98373442** |
|  |  | **7569** | **24** | **0.017** | **0.8956926** |
|  | **965** | **7088** | **30** | **1.04** | **0.30690234** |
|  |  | **7089** | **30** | **3.53** | **0.06026931** |
|  | **966** | **7572** | **37** | **58.06** | **1E-12** |
|  |  | **7573** | **35** | **32.78** | **1.0298E-08** |
|  | **967** | **7577** | **36** | **42.69** | **6.4E-11** |
|  |  | **7579** | **31** | **12.8** | **0.00034734** |
|  | **968** | **7584** | **19** | **51.29** | **1E-12** |
|  |  | **7586** | **25** | **18.85** | **1.4104E-05** |
|  | **970** | **7056** | **37** | **33.16** | **8.467E-09** |
|  |  | **7058** | **32** | **18.42** | **1.7727E-05** |
|  | **972** | **7587** | **37** | **50.21** | **1E-12** |
|  |  | **7588** | **31** | **18.83** | **4.0928E-05** |
|  | **973** | **7596** | **34** | **22.77** | **1.8266E-06** |
|  |  | **7597** | **30** | **7.08** | **0.00777523** |
|  | **974** | **7599** | **31** | **11.68** | **0.00063028** |
|  |  | **7600** | **29** | **0.53** | **0.46552149** |
|  | **975** | **7603** | **29** | **5.19** | **0.02277659** |
|  |  | **7604** | **37** | **60.66** | **1E-12** |
|  | **976** | **7607** | **31** | **5.72** | **0.01672712** |
|  |  | **7608** | **28** | **1.66** | **0.19714333** |
|  | **977** | **7614** | **25** | **9.41** | **0.00215975** |
|  |  | **7616** | **24** | **0.003** | **0.95798083** |
|  | **978** | **7620** | **14** | **82.31** | **1E-12** |
|  |  | **7622** | **18** | **58.61** | **1E-12** |
|  | **979** | **7630** | **33** | **13.45** | **0.00024539** |
|  | **980** | **7637** | **22** | **33.37** | **7.621E-09** |
|  |  | **7638** | **24** | **28.71** | **8.3858E-08** |
|  | **981** | **7050** | **22** | **0.98** | **0.32244099** |
|  |  | **7053** | **23** | **0.005** | **0.94385681** |
|  | **982** | **7639** | **29** | **0.23** | **0.63468758** |
|  |  | **7642** | **36** | **34.04** | **5.407E-09** |
|  | **983** | **7649** | **31** | **2.97** | **0.08500427** |
|  |  | **7650** | **32.5** | **17.04** | **3.6635E-05** |
|  | **984** | **7652** | **34** | **36.05** | **1.927E-09** |
|  |  | **7653** | **31** | **11.14** | **0.00084534** |
|  | **985** | **7664** | **30** | **10.05** | **0.00152625** |
|  |  | **7665** | **33** | **22.74** | **1.8569E-06** |
|  | **986** | **7667** | **34** | **28.35** | **1.0127E-07** |
|  |  | **7668** | **34** | **33.09** | **8.794E-09** |
|  | **987** | **7672** | **28** | **0.43** | **0.5103775** |
|  |  | **7673** | **31** | **6.38** | **0.0115661** |
|  | **988** | **7083** | **30** | **4.74** | **0.02940857** |
|  | **989** | **7674** | **18** | **60.78** | **1E-12** |
|  |  | **7677** | **23** | **27.98** | **1.2282E-07** |
|  | **990** | **7679** | **33** | **22.25** | **2.3879E-06** |
|  |  | **7682** | **36** | **44.61** | **2.4E-11** |
|  | **991** | **7684** | **33** | **12.14** | **0.00049244** |
|  | **992** | **7690** | **24** | **15.61** | **7.8002E-05** |
|  | **993** | **7700** | **24** | **20.04** | **7.5673E-06** |
|  |  | **7701** | **36.5** | **47.54** | **5E-12** |
|  | **995** | **7720** | **24** | **14.1** | **0.00017291** |
|  | **996** | **7721** | **33** | **12.5** | **0.00040748** |
|  |  | **7722** | **24** | **4.96** | **0.02593473** |
|  | **997** | **800** | **28** | **0.002** | **0.95942718** |
|  |  | **801** | **28** | **4.98** | **0.02560103** |
|  |  | **804** | **24.5** | **0.46** | **0.49718284** |
|  |  | **807** | **28** | **11.87** | **0.00056906** |
|  | **998** | **7726** | **28** | **5.07** | **0.02430675** |
|  | **999** | **7734** | **28** | **1.27** | **0.25946132** |
|  |  | **7735** | **24** | **4.28** | **0.03858767** |
|  | **1000** | **7403** | **30.5** | **9.35** | **0.00222944** |
|  |  | **7404** | **28** | **1.22** | **0.27031886** |
|  | **1001** | **7408** | **27** | **0.11** | **0.74268531** |
|  |  | **7409** | **32** | **21.58** | **3.3857E-06** |
|  | **1002** | **7413** | **29** | **12.03** | **0.00052258** |
|  |  | **7414** | **32** | **13.47** | **0.00024246** |
|  | **1003** | **7423** | **32** | **3.99** | **0.04578995** |
|  |  | **7424** | **37** | **51.06** | **1E-12** |
|  | **1004** | **7428** | **29** | **0.6** | **0.43868314** |
|  | **1005** | **7429** | **26** | **0.14** | **0.70640412** |
|  | **1006** | **7430** | **26** | **4.89** | **0.02706067** |
|  |  | **7431** | **29** | **1.34** | **0.24675806** |
|  | **1007** | **7432** | **36** | **34.08** | **5.291E-09** |
|  | **1008** | **7434** | **32** | **9.53** | **0.00202307** |
|  |  | **7435** | **36** | **32.84** | **1.0012E-08** |
|  | **1009** | **7442** | **36** | **27.89** | **1.2854E-07** |
|  |  | **744X** | **33** | **24.82** | **6.2795E-07** |
|  | **1010** | **7449** | **33** | **13.8** | **0.00020359** |
|  | **1012** | **7450** | **36** | **47.59** | **5E-12** |
|  |  | **7455** | **33** | **26.04** | **3.3524E-07** |
|  | **1013** | **7456** | **29** | **2.51** | **0.11341366** |
|  |  | **7457** | **32** | **17.07** | **3.6017E-05** |
|  | **1014** | **7458** | **30** | **21.17** | **7.0944E-06** |
|  |  | **7459** | **30** | **0.47** | **0.49316792** |
|  | **1015** | **7464** | **26** | **0.64** | **0.4254404** |
|  |  | **7465** | **21.5** | **7.07** | **0.00783017** |
|  | **1017** | **7468** | **30** | **2.47** | **0.11618628** |
|  |  | **7469** | **36** | **37.39** | **9.69E-10** |
|  | **bantam** | **6542** | **28** | **2.39** | **0.12206605** |
